# A possible combined appraisal pattern: predicting the prognosis of patients after esophagectomy

**DOI:** 10.1186/s12957-023-03020-x

**Published:** 2023-05-22

**Authors:** LiangLiang Chen, GuoCan Yu, WuChen Zhao, Bo Ye, YuSheng Shu

**Affiliations:** 1 Department of Thoracic Surgery, Affiliated Hangzhou Chest Hospital, Zhejiang University School of Medicine, Hangzhou, 310005 China; 2 Department of Thoracic Surgery, Northern Jiangsu People’s Hospital, Clinical Medical School of, Yangzhou University, Yangzhou, 225001 China

**Keywords:** Esophageal carcinoma, Nutritional status, Inflammatory response, Prognosis, Prediction model

## Abstract

**Objective:**

To investigate the predictive merit of combined preoperative nutritional condition and systemic inflammation on the prognosis of patients receiving esophagectomy, with the assessment of model construction to extract a multidisciplinary phantom having clinical relevance and suitability.

**Methods:**

The software of R 4.1.2 was utilized to acquire the survival optimal truncation value and the confusion matrix of survival for the continuity variables. SPSS Statistics 26 was employed to analyze the correlation of parameters, where including *t*-test, ANOVA and the nonparametric rank sum test shall. Pearson chi-square test was used for categorical variables. The survival curve was retrieved by Kaplan–Meier method. Univariate analysis of overall survival (OS) was performed through log-rank test. Cox analysis was for survival analyze. The performance of the prediction phantom through the area under curve (AUC) of receiver operating characteristic curve (ROC), decision curve analysis (DCA), nomogram and clinical impact curve (CIC) was plotted by R.

**Results:**

The AUC value of albumin-globulin score and skeletal muscle index (CAS) is markedly superior. Patients with diminished AGS and greater SMI were associated with improved overall survival (OS) and recurrence-free survival (RFS) (*P* < 0.01). The CAS composite evaluation model was calibrated with better accuracy and predictive performance. The DCA and CIC indicated a relatively higher net revenue for the prediction model.

**Conclusions:**

The prediction model including the CAS score has excellent accuracy, a high net revenue, and favorable prediction function.

**Supplementary Information:**

The online version contains supplementary material available at 10.1186/s12957-023-03020-x.

## Introduction

Esophageal cancer, one of the most aggressive gastrointestinal malignancies to date and currently the sixth leading cause of cancer-associated death, is as yet experiencing an increasing trend in incidence [1]. Based on the Global Cancer Report 2020, 604,100 cases of esophageal cancer were diagnosed worldwide in 1 year, and 544,076 of them occurred due to esophageal cancer [2]. China is one of the most prevalent regions of esophageal cancer in Asia, and according to the China Cancer Data Center, there were 246,000 cases of esophageal cancer diagnosis in China in 2015, and 188,000 of them died from esophageal cancer [3]. Consequently, the identification of credible biomarkers to forecast patients’ high risk of recurrence as well as to institute an even more effectively therapeutic strategy has thus now emerged as a high-priority field in the esophageal cancer theater community.

The clinical manifestations of esophageal cancer patients are inevitably accompanied by varying degrees of malnutrition due to anorexia, dysphagia, or even mechanical obstruction, which means they may also undergo skeletal muscle exertion, which is a substantial component of the cachexia of cancer as well as another essential pathological alteration in the development of carcinoma, with considerable potential to affect the survival outcome of patients [4]. In recent years, it has already been documented that skeletal sarcopenia and its indexes may affect the prognosis of patients with various carcinomas, including those of the gastrointestinal system [5]. In addition, it has been demonstrated that the systemic inflammatory response plays an overwhelming influence on host metabolism during carcinoma progression and may lead to catabolism of skeletal muscle, while muscle depletion invites further evolution of the inflammatory response, which in turn triggers a vicious loop that expends further muscle [6]. Thereby, systemic inflammatory response and malnutrition can mutually attack the tumor microenvironment and damage immune function, inviting angiogenesis and immune escape, both of which have a major contribution in tumor progression [7]. Thereby, ordinary indicators of inflammatory index including neutrophil-to-lymphocyte ratio (NLR) [8], platelet-to-lymphocyte ratio (PLR) [9], modified Glasgow prognostic score [10], and other multiple models, as well as skeletal muscle mass index (SMI) associated with NLR, had been investigated in prior studies [11]. This demonstrates that the system-wide inflammatory response and malnutrition can indeed cooperate to compromise the prognosis of individual patients with cancer. Subsequently, the investigators presented a novel metric, albumin-globulin score and skeletal muscle index (CAS), incorporating indicators of nutritional status and inflammation, to facilitate comparison between the metrics to predict postoperative survival. While there is not yet a correlative study to assess the prognostic evaluation of CAS on patients with esophageal cancer after surgery, we proposed CAS to investigate the practicality in forecasting the prognosis of patients after radical resection of esophageal cancer in terms of predictability and clinical application.

## Materials and methods

### Patient

A retrospective study was performed to enroll 256 patients with postoperative stages 1–3 esophageal squamous cell carcinoma in the Department of Thoracic Surgery of Subei People’s Hospital from January 1, 2016, to February 31, 2017.

Patient inclusion criteria are as follows: (1) patients with esophageal cancer diagnosed by imaging techniques such as endoscopy or CT from January 1, 2016, to February 31, 2017, without distant metastasis; (2) patients with postoperative pathology of stages 1 to 3 esophageal squamous cell carcinoma (according to the 8th version of TNM staging guidelines); (3) no previous experience of second primary tumor, no chronic wasting diseases, and no rheumatic immunological diseases; (4) the first therapeutic protocol was radical surgical resection, with no other preoperative treatment, even if postoperative adjuvant therapy was required; and (5) the data were complete and available. A complete postoperative follow-up data is available.

### Follow-up time

 From the initiation of operation until March 1, 2022, or the final event (death), with the ending of overall survival at the time of the patient’s death due to esophageal cancer-related death. During the period, all postoperative patients will be scheduled for regular outpatient follow-up at least once every 3 months to 6 months after 3 years postoperatively and at minimum once a year after 5 years postoperatively.

### Statistical analysis

The present figures consist of continuous as well as categorical variables. For the former, we choose the median and interquartile range or the mean ± standard deviation to represent the distribution; as for the categorical variables, we opt for the proportion of n% to denote the spread. The R language 4.1.2 software was implemented to obtain the survival optimal truncation values and confusion matrix for continuous variables. SPSS statistics.26 software was applied for statistical analysis of parametric correlations, in which the independent sample *t*-test method or univariate ANOVA or the nonparametric rank-sum test was used for continuous variables, and the Pearson chi-square test was performed for categorical variables. Survival profiles were analyzed via Kaplan–Meier method, and univariate analysis of postoperative OS rate was performed by log-rank test, with factors included in Cox analysis when *P* < 0.2. The ROC in terms of AUC, C-index, DCA, nomogram, and CIC were constructed by R. For the difference, *P* < 0.05 was interpreted as statistically significant.

### Data processing

According to the Clavien-Dindo scoring system for postoperative complications, they were defined as none or mild, II, IIIa, and IIIb as medium, and IVa and IVb as severe [12].

SMI was calculated by manually outlining the area of all skeletal muscle tissue at the L3 level in the CT of the upper abdomen divided by the square of the height, and the optimal cut-off values were obtained based on gender grouping and then divided into high-risk and low-risk groups based on the cut-off values. A qualified and trained imaging physician blindly measured all patient figures: CIN: [SMI (cm^2^/m^2^) × serum albumin (g/dL)]/NLR [13]. Albumin-globulin score (AGS) was defined as follows: patients (albumin values > 41.7 g/L and globulin ≤ 28.6 g/L) were having a “0”; hypoalbuminemia (≤ 41.7 g/L) and high globulin levels (> 28.6 g/L) were classified as “2”; and the remaining were “1” [14]. Patients with simultaneous low AGS and low-risk SMI were further subdivided into “1”; patients with high AGS (1/2) and high-risk SMI were categorized into “3”; and the rest were apportioned into “2” [15].

## Results

### Correlation analysis

**Fig. 1:**
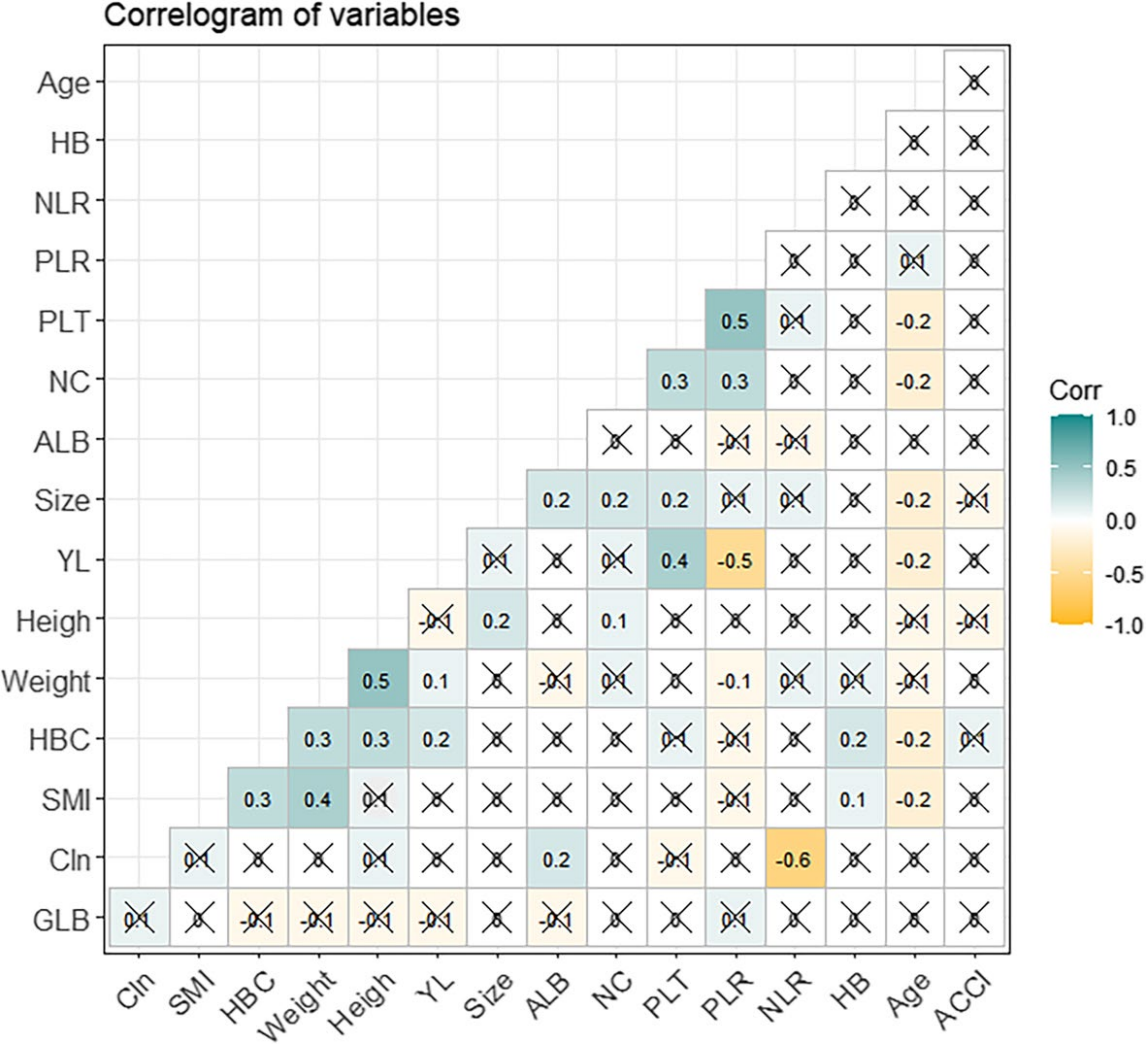
Correlation matrix of perioperative nutritional and inflammatory indicators

The indicators with statistical differences are based on the above analysis. Among them, the SMI was strongly correlated with gender (*P* = 0.03) as well as the CIN, as shown in Fig. 1. For continuous variables, it was evident that the SMI were positively corroborated with HBC (*r* = 0.3) and weight (*r* = 0.4) and negatively with age (*r* =  − 0.2). In contrast, there was no correlation between SMI, NLR, and PLR, so as to the albumin and globulin. In any case, no correlation was found between CIN and SMI, NLR, and ALB content.

### Pertinence analysis of CAS and clinicopathological characteristics

**Fig. 2:**
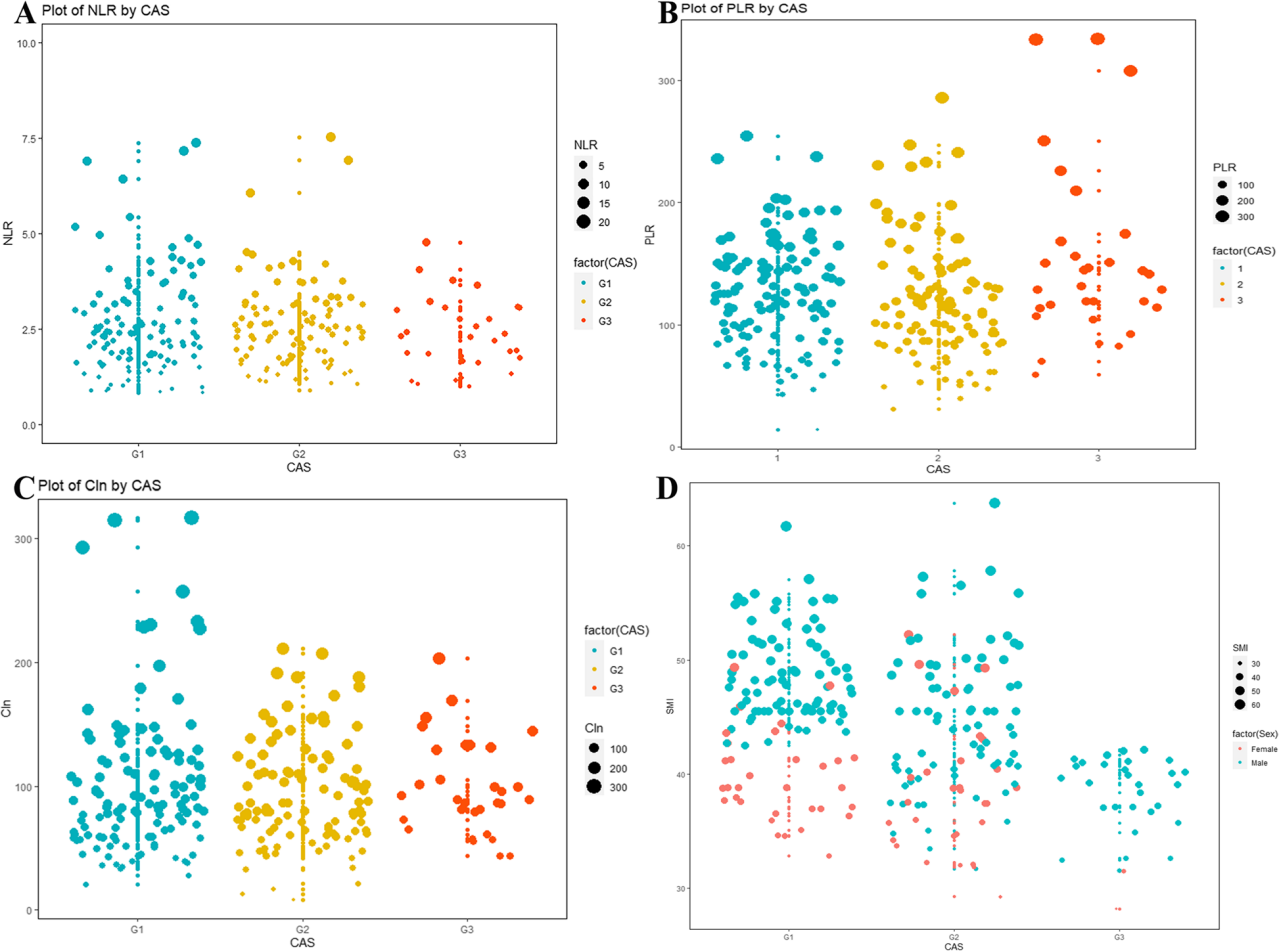
Correlation analysis and distribution of CAS indicators. **A** CAS and NLR. **B** CAS and PLR. **C** CAS and CIN. **D** CAS gender-related SMI

A total of 256 patients with stages 1–3 esophageal squamous cell carcinoma were ultimately entered into this study according to the inclusion criteria. Among them, 63 patients (75.4%) were female, and 193 patients (24.6%) were male, with a median age of 65 years and an age quartile of 62–69 years. Patients were scanned in detail by combining the age-adjusted Charlson Comorbidity Index (ACCI) scores [16, 17]. Combining the SMI and AGS, we could enumerate 118 cases (46.1%) in G1; 107 cases (41.8%) in the G2; and 31 cases (12.1%) in the G3, and all of them were statistically different (*P* < 0.001). The CAS was tangentially correlated with patient age (*P* = 0.03, *F* = 3.4), gender (*P* = 0.04, *χ*^2^ = 6.27, *V* = 0.2), alcohol consumption (*P* = 0.04, *χ*^2^ = 6.5, *V* = 0.2), the mean lymphocyte count (*P* < 0.01, *F* = 6.8), NLR (*P* = 0.03, *F* = 3.7), PLR (*P* = 0.04, *F* = 5.6), and CIN (*P* = 0.05, *F* = 3.1), as shown in Table 1 and Fig. 2.

**Table 1:** Correlation analysis of clinicopathological features and CAS indicators

Variable	G1	G2	G3	*P*	*χ*^2^/F	V
Age (y)	64.1 ± 6.0	65.1 ± 5.4	67 ± 6.7	0.03	3.4	*
Sex						
Male	87 (73.1%)	77 (72.6%)	29 (93.5%)	0.04	6.3	0.2
Female	32 (26.9%)	29 (27.4%)	2 (6.5%)			
Drinking						
No	79 (66.4%)	68 (64.2%)	13 (41.9%)	0.04	6.5	0.2
Yes	40 (33.6%)	38 (35.8%)	18 (58.1%)			
ACCI						
1–2	38 (31.9%)	34 (32.1%)	10 (32.3%)	0.98	0.5	0.03
3–4	67 (56.3%)	58 (54.7%)	16 (51.6%)			
5–7	14 (11.8%)	14 (13.2%)	5 (16.1%)			
Location						
Up	10 (8.4%)	7 (6.6%)	4 (12.9%)	0.73	2.0	0.1
Med	75 (63.0%)	71 (67.0%)	17 (54.8%)			
Low	34 (28.6%)	28 (26.4%)	10 (32.3%)			
Size (cm)	4.4 ± 1.6	4.8 ± 1.5	5.0 ± 2.0	0.12	2.1	*
P-TNM						
I	46 (38.7%)	35 (33.0%)	9 (29.0%)	0.45	3.7	0.1
II	45 (37.8%)	35 (33.0%)	13 (41.9%)			
III	28 (23.5%)	36 (34.0%)	9 (29.0%)			
NC (10^9^/L)	3.7 ± 1.4	4.2 ± 1.7	4.3 ± 2.4	0.07	2.7	*
LY (10^9^/L)	1.5 ± 0.5	1.8 ± 0.7	1.6 ± 0.7	0.00	6.8	*
PLT (10^9^/L)	184.4 ± 61.7	199.9 ± 70.8	210.2 ± 74.9	0.08	2.5	*
NLR	2.6 ± 1.2	2.6 ± 1.5	3.5 ± 3.9	0.03	3.7	*
CIN	99.1 ± 41.6	99.7 ± 57.6	75.9 ± 42.5	0.045	3.1	*
PLR	124.9 ± 43.3	118.7 ± 48.8	152.6 ± 71.0	0.04	5.6	*

*null

### ROC curve specifies the relevant threshold

**Fig. 3:**
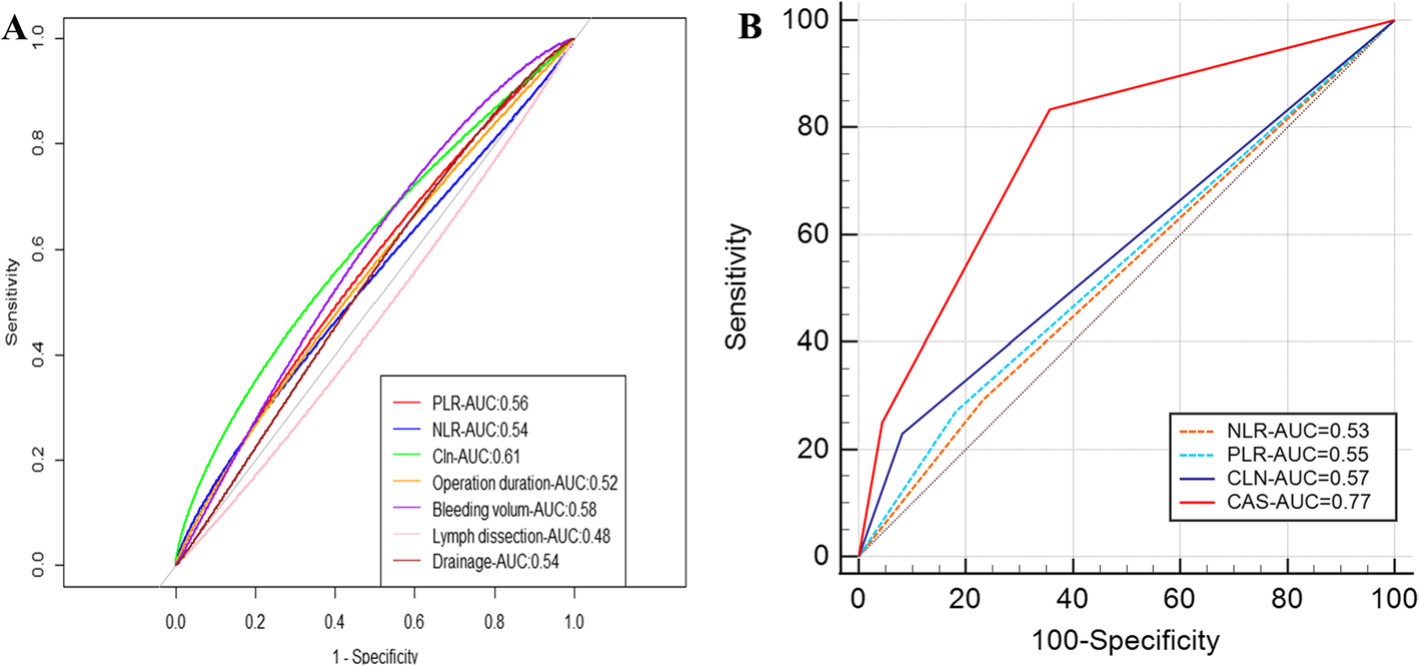
**A** ROC curve of each variable and corresponding AUC value. **B** ROC curve and value of PLR, NLR, CIN, and CAS comprehensive scoring index

The AUC, accuracy, and sensitivity of each continuous variable calculated by our plotted ROC curves are detailed in Fig. 3. The optimal cut-off value of SMI for females was 32.9, with an AUC area of 0.73, sensitivity of 45.0%, and accuracy of 44.0%, while for males was 42.3, with an AUC area of 0.70, sensitivity of 22.0%, and accuracy of 25.0%. Subsequently, we proceeded to plot the area under the curve of the ROC curve pertaining to the categorical variables of the four indicators of PLR, NLR, CIN, and CAS, and the evaluation showed that the AUC value of CAS was significantly better than the NLR (*AUC* = 0.53), PLR (*AUC* = 0.55), and CIN (*AUC* = 0.57).

### Survival analysis

The median follow-up of patients in this study was 63 months (interquartile: 60 to 68 months), of which 192 patients were kept alive at the end of follow-up. The 5-year OS and DFS rates were calculated to be 76.9% and 86.5%, respectively.

**Fig. 4:**
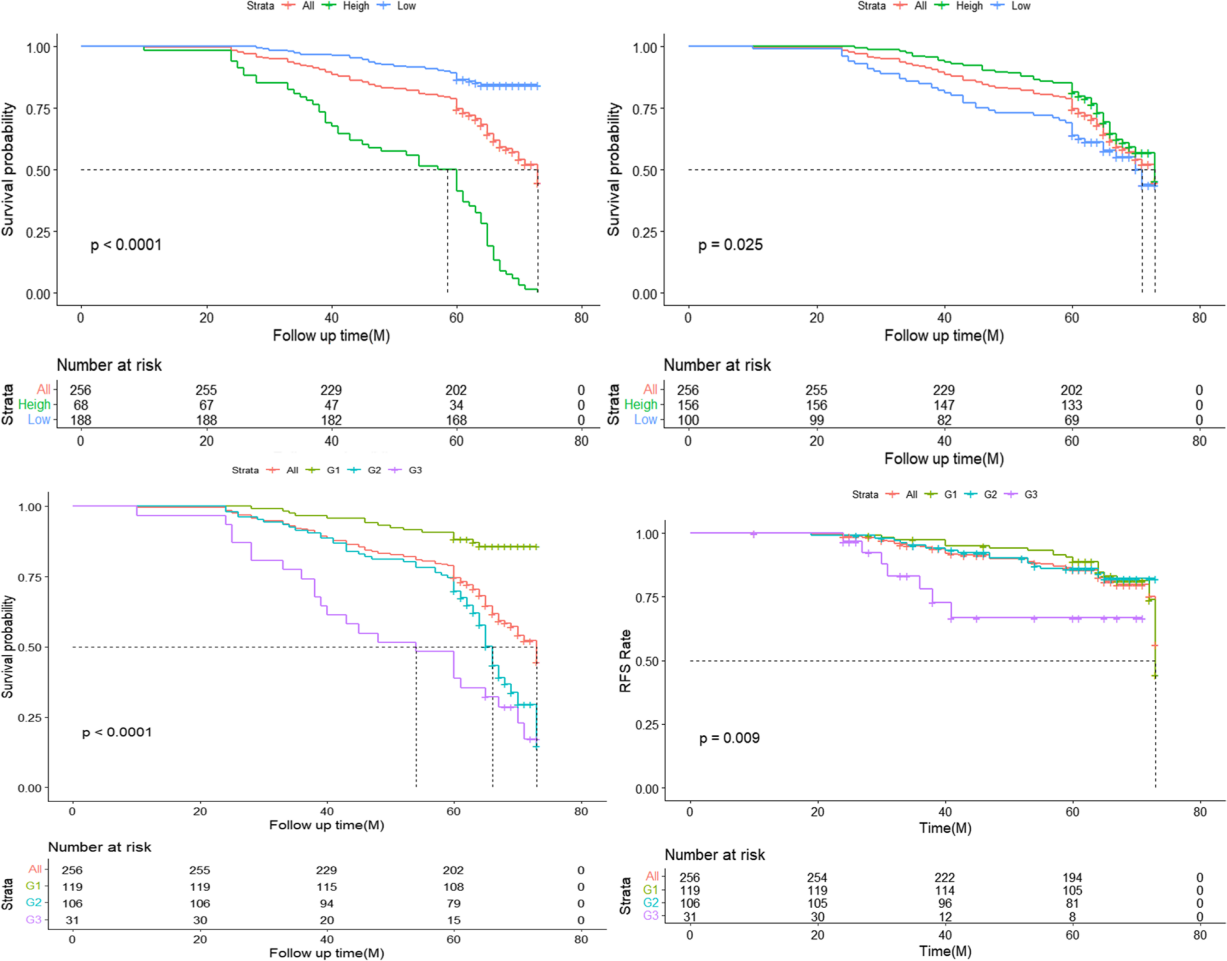
**A** K-M curves of correlation analysis between SMI and survival in high-risk and low-risk groups. **B** K-M curves of survival correlation analysis between high-risk and low-risk groups of AGS. **C** CAS overall survival curve. **D** CAS RFS rate curve

In the results of univariate analysis, detailed in Table 2, all visual variables with *P* < 0.2 were enrolled for final analysis by further COX regression. Among them, positive postoperative recurrence and metastasis (*HR* = 2.15, *CI* = 1.16–4.00, *P* = 0.02) and CAS were investigated as independent factors affecting patients’ prognosis. However, TNM stage, chemoradiotherapy, and CAS score were independent prognostic factors for DFS (*P* < 0.05). Moreover, CAS is as well an individual element that influences the postoperative RFS of patients. In addition, the prognosis of patients in the “2” group (*HR* = 4.77, *CI* = 2.59–8.80, *P* < 0.01) and the “3” group (*HR* = 8.36, *CI* = 4.19–16.67, *P* < 0.01) was also worse than that of patients in the “1” group, and the hazard of death was fourfold higher in the “2” group and eightfold higher in the “3” group than in the “1” group. Patients with “3” group (*HR* = 4.75, *CI* = 1.78–12.69, *P* < 0.01) and “2” group (*HR* = 1.75, *CI* = 1.38–1.48, *P* < 0.01) were estimated to have worse DFS than patients with low-risk factors. In conclusion, the K-M curves are pertaining to SMI, AGS, and CAS, as detailed in Fig. 4.

**Table 2:** Univariate and multifactorial analysis in 256 cases of esophageal cancer

Variable	Overall survival				Recurrence-free survival			
	Univariate		Multivariate		Univariate		Multivariate	
	HR (95% *CI*)	*P*	HR (95% *CI*)	*P*	HR (95% *CI*)	*P*	HR (95% *CI*)	*P*
Age (years)	1.03 (0.99–1.07)	0.10	1.04 (0.99–1.09)	0.10	0.99 (0.95–1.05)	0.95		
Sex (female vs male)	1.69 (0.99–2.89)	0.06	1.38 (0.74–2.58)	0.31	2.66 (1.05–6.77)	0.04	1.56 (0.59–4.11)	0.37
ACCI		0.16				0.72		
3–4 vs 1–2	0.98 (0.62–1.54)	0.92	1.02 (0.61–1.72)	0.93	0.82 (0.43–1.57)	0.55		
5–7 vs 1–2	1.67 (0.91–3.06)	0.10	1.58 (0.75–3.32)	0.23	1.13 (0.44–2.90)	0.80		
Size (cm)	1.18 (1.04–1.32)	0.01	1.07 (0.93–1.24)	0.35	1.20 (1.01–1.43)	0.04	0.84 (0.67–1.06)	0.14
TNM		0.00				0.00		**0.01**
II vs I	1.14 (0.67–1.92)	0.64	1.03 (0.58–1.86)	0.91	1.06 (0.43–2.62)	0.90	0.34 (0.12–0.99)	0.04
III vs I	2.24 (1.36–3.66)	0.00	1.56 (0.82–2.95)	0.17	4.24 (1.98–9.10)	0.00	1.22 (0.47–3.19)	0.69
Bleeding (< 140 vs ≥ 140)	1.41 (0.94–2.12)	0.10	1.07 (0.67–1.69)	0.79	1.54 (0.85–2.80)	0.16	1.45 (0.78–2.71)	0.24
Vascular invasion (no vs yes)	2.14 (1.19–3.84)	0.01	1.45 (0.75–2.80)	0.27	2.43 (1.08–5.47)	0.03	0.85 (0.33–2.23)	0.75
Complications		0.08				0.04		0.36
Mild vs no	0.73 (0.44–1.19)	0.20	1.04 (0.60–1.81)	0.88	1.24 (0.62–2.46)	0.54	1.12 (0.53–2.35)	0.78
Severe vs no	1.56 (0.88–2.75)	0.13	1.40 (0.75–2.64)	0.29	2.76 (1.26–6.02)	0.11	2.91 (1.28–6.62)	**0.01**
Chemoradiotherapy (no vs yes)	1.60 (1.07–2.40)	0.02	0.86 (0.48–1.55)	0.62	8.36 (4.01–17.42)	0.00	12.69 (4.75–33.90)	**0.00**
Metastasis (no vs yes)	2.34 (1.50–3.66)	0.00	2.15 (1.16–4.00)	**0.02**				
NC (10^9^/L)	1.17 (1.04–1.31)	0.01	1.02 (0.86–1.20)	0.86	1.12 (0.94–1.34)	0.22		
Hospital stay (D)	1.03 (0.99–1.07)	0.16	1.02 (0.99–1.06)	0.25	1.03 (0.97–1.10)	0.34		
PLR (< 154.9 vs ≥ 154.9)	1.55 (0.99–2.43)	0.06	1.42 (0.80–2.51)	0.23	1.45 (0.74–2.83)	0.27		
SMI (height vs low)	0.09 (0.06–0.14)	0.00			0.62 (0.32–1.21)	0.16		
CIN (< 51.6 vs ≥ 51.6)	0.43 (0.27–0.69)	0.00	0.90 (0.43–1.89)	0.77	0.83 (0.35–1.98)	0.69		
CAS		0.00		**0.00**		0.02		**0.00**
G2 vs G1	4.96 (2.84–8.86)	0.00	4.77 (2.59–8.80)	0.00	1.97 (1.50–1.86)	0.02	1.75 (1.38–1.48)	0.01
G3 vs G1	8.60 (4.57–16.21)	0.00	8.36 (4.19–16.67)	0.00	3.31 (1.83–7.92)	0.01	4.75 (1.78–12.69)	0.00

### Model construction

**Fig. 5:**
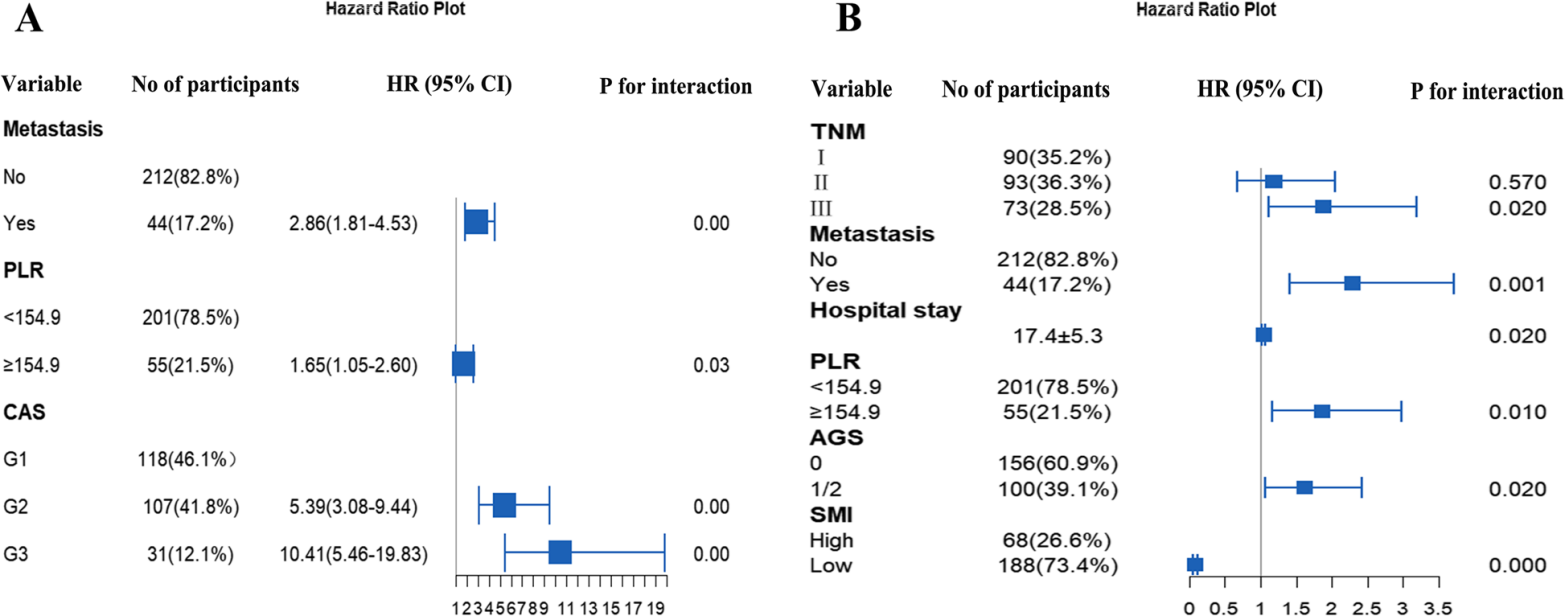
COX analysis to construct prediction model visualization forest map. **A** Model A. **B** Model B

Based on the above analysis, we continued to carry out the indicators that have significant impact on postoperative survival of patients in our study, as adjustment conditions, and then constructed relevant prediction models. The results showed that, only the score of CAS, SMI combined with AGS could be independent factors affecting the survival of patients after esophageal cancer surgery. Subsequently, the relevant model visualization forest map was then plotted. Among them, model A demonstrated that the CAS had the most significant impact on the OS rate of patients, followed by postoperative tumor metastasis or recurrence, and the least of PLR, while model B showed that SMI most markedly affected patients’ postoperative survival, and AGS had the least ability to influence, respectively, as detailed in Fig. 5.

### Validation and application

**Fig. 6:**
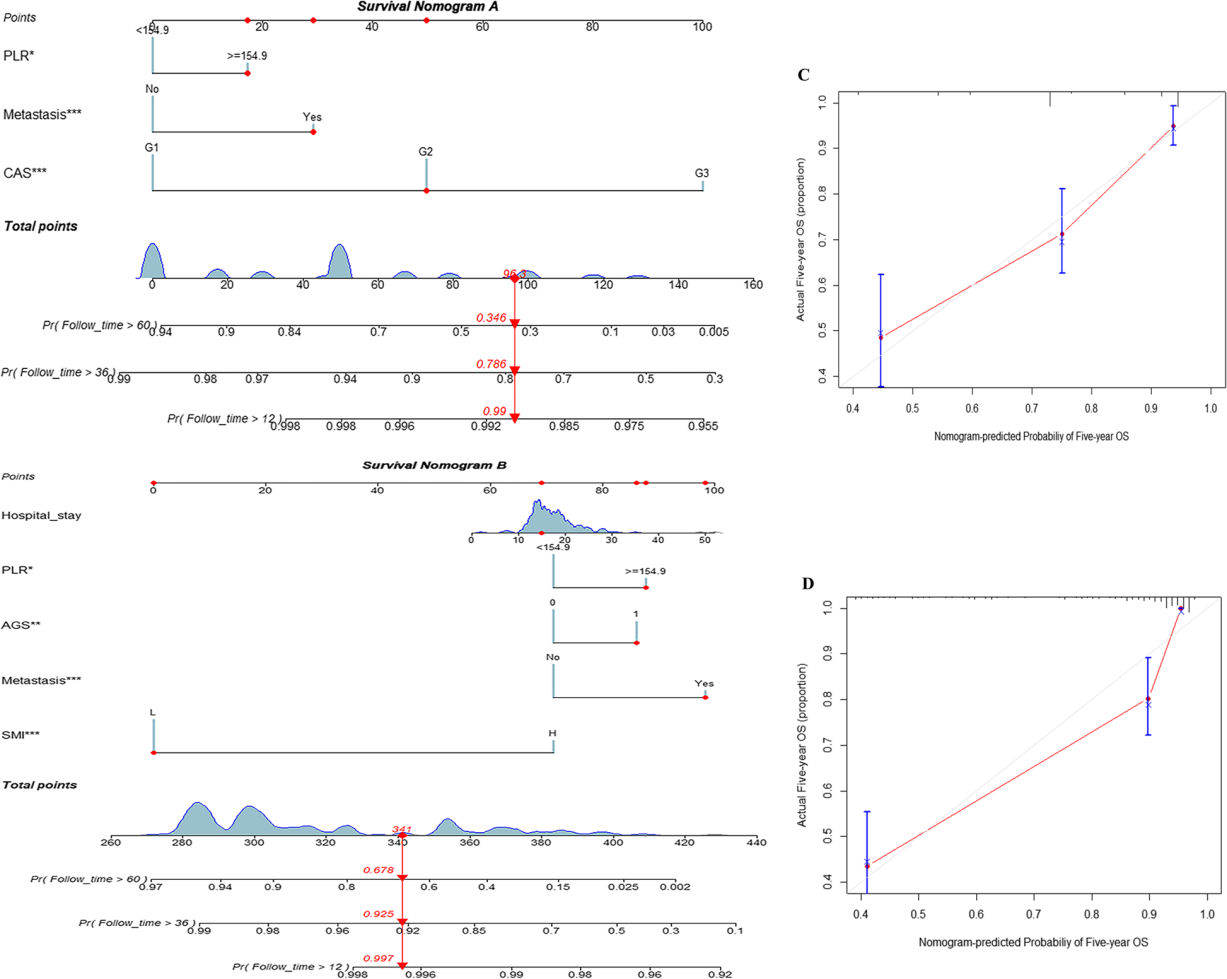
Nomogram of predicting 1-, 3-, and 5-year survival probabilities after esophagectomy. **A** CAS adjustment model. **B** SMI combined with AGS to adjust the model. **C** Calibration curve after 5 years of model A. **D** Calibration curve after 5 years of model B

**Fig. 7:**
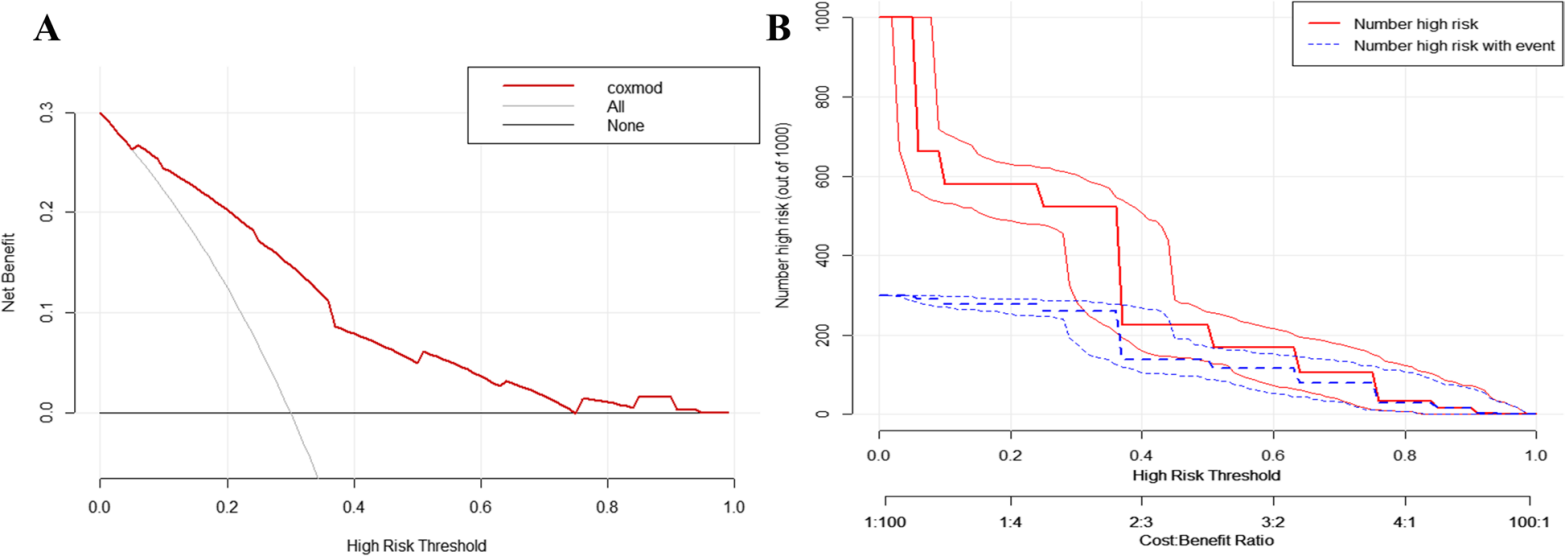
Model A-related evaluation and clinical benefit analysis. **A** Decision curve analysis (DCA). **B** Clinical impact curve (CIC)

The C-index results of model A fluctuated from 0.7 to 0.8, and model B floated from 0.8 to 0.9. After that, calibration curves showing that the model had an excellent accuracy of calibration. Figure 6 displays that the nomogram and the predicted 5-year survival of model A and model B suggested that the two models are consistent, and the accuracy of the model A calibration is favorable. Following the process of constructing clinical decision curves, the outcomes could be derived that the prediction model A had a high net benefit throughout the interval, indicating that the model has a superior clinical application merit for the prediction of prognostic survival of esophageal cancer patients. Meanwhile, the clinical impact curve visually illustrates that the model A has superior overall net return within the extent of the threshold probability and significantly affects patient prognosis, which points to the well-predicted merit of the model, as detailed in Fig. 7.

## Discussions

In more than a decade, sequelae of patients with esophageal cancer have remained poor in terms of prognosis, in spite of improvements in operative techniques, rational lymph node dissection, and aggressive treatment strategies [18]. To that end, further elucidation of pathogenesis and exploration of possible hazard factors are essential for early target screening of high-risk populations [14].

Anecdotal studies have revealed that the majority of patients are frequently associated with systemic inflammation, persistent muscle, and weight reduction, which are typically considered hallmark features of cachexia [19]. The consequent deterioration in nutritional profile leads to cachexia, with further aggravation of weight loss and reduction in skeletal muscle mass [20]. Among them, a prospective study by Arfon Powell et al. [21] demonstrates that SMI are an independent prognostic factor affecting patients with esophageal cancer after operation and have a promising potential. Jessie A. Elliott et al. [22] evidence that increased skeletal sarcopenia is notably associated with an increased risk of serious postoperative complications. Reut Anconina et al. [23] had shown that sarcopenia can markedly alter the overall prognostic value of patients after operation. Yusuke Ishibashi et al. [24] further integrated analysis by meta-analysis established a robust correlation between poor prognosis and NLR in patients with esophageal cancer. Juhong Deng et al. [25] suggest that elevated PLR are dramatically linked to worse prognosis in esophageal carcinoma and tightly associated with poorer clinicopathological characteristics. In additions, another proposal has been made to present the AGS as an alternative prognostic model to anticipate the prognosis of certain tumors [26]. Bearing in mind that both AGS and SMI, as well as NLR and PLR, have an instrumental role in the prognosis of patients with esophageal cancer, the investigators introduced a novel index (CAS classification) that combines indicators of nutritional status and inflammation to predict postoperative survival by comparing between the indicators. Weipu Mao et al. [27] identified that CAS as an individual prognostic risk factor for OS and CSS in patients with renal cell carcinoma undergoing nephrectomy and was considered superior to AGS and SMI.

In our current study, we present an assessment of the financial and prognostic worth of 256 patients with esophageal carcinoma. The findings of the univariate analysis revealed that the tumor size, postoperative pathologic p-TNM, vascular invasion, postoperative radiotherapy, recurrence or metastasis, neutrophil count, CIN, and CAS were capable of significantly affecting the postoperative prognostic survival of patients with esophageal carcinoma. Nevertheless, gender, tumor size, p-TNM, vascular invasion, postoperative complications, radiotherapy, and CAS were intimately associated with RFS rates. The ROC evaluation showed that the AUC value of CAS was significantly better than the NLR, PLR, and CIN. Further multivariate regression demonstrated that patients with positive postoperative recurrence and metastasis, and CAS were independent variables affecting patient prognosis. Moreover, patients with diminished AGS and increased SMI were associated with improved overall survival (OS) and recurrence-free survival (RFS). When we adjusted the case grouping according to CAS, we found that the CAS was capable of expanding the predictive ability of nutritional index SMI or CIN and inflammatory index NLR or PLR, substituting to anticipate postoperative survival in esophageal cancer, and it was more representative. Subsequently, the outcomes demonstrated that either the AGS, CIN, SMI, and PLR or CAS had an influence on the prognosis of patients, except that ultimately the CAS and the SMI combined with the AGS could be independent factors affecting the survival of patients, whose C-index indicated that the two models had superior prediction accuracy, with model A having 70–80% certainty, while model B had 80–90% certainty in confirming the OS rate of esophageal cancer patients at 5 years postoperatively to be 76.9%. In our present study, the RFS curve of patients in the G3 group was found to be markedly superior to the OS yield, which may be attributed to the possibility that the group is a high-risk category, both in terms of nutritional status and immune response, with the majority of patients dying from postoperative cachexia or nutritional depletion, as well as a frequent cause of death in patients with esophageal cancer after surgery. In addition, an RFS curve for the G3 group showed that most of the patients died of cachexia rather than metastasis after 5 years of follow-up. Of course, it is not representative of the results obtained, since many advanced carcinoma patients in China are not willing to undergo effective comprehensive examinations, as many patients die during the follow-up process without being able to eliminate the possibility of metastasis, thus a large amount of multi-center studies are needed to verify the results later. Therefore, two models were constructed for correlative comparison and validation. The data indicate that the models calculated from the model A and model B were consistent in predicting the actual survival at 5 years, meaning that two models were comparable, and the CAS integrated evaluation model was more calibrated in terms of accuracy. In a further step, we reviewed the CAS, NLR, PLR, and CIN as indices by comparing the AUC of ROC curves, and the CAS proved to be predictive of superior outcomes. In our study, although CIN, NLR and PLR have been demonstrated to serve an essential role in the prognostic assessment of patients with esophageal cancer undergoing operation, none of them was ascertained to be an isolated predictor of prognosis in patients. In a way, this implies that the CAS performs a major role in the assessment of ESCC patients than other inflammation-related etiologies. In the same vein, the prediction model was constructed based on the CAS, and the correlation column line graph was plotted. The model calibration illustrated that the accuracy of the model calibration was favorable. DCA and CIC revealed a superior net return for this prediction model. Consequently, the CAS is suitable for comprehensive evaluation of nutritional situation and systemic inflammation in patients with esophageal cancer and is valuable for clinical utilization as well as having an influential effect on the survival of patients with excellent accuracy.

In contrast to other serologic diagnostic or imaging assays, ALB, GLB and skeletal muscle mass are all routinely performed in operated patients and are accessible to calculate. In addition, these parameters are low-cost and do not increase total healthcare expenses. Moreover, our study found that CAS grading integrates the preoperative nutritional status and systemic inflammation of patients, which may amplify the predictive accuracy of ALB, GLB, and SMI in terms of prognosis. In some ways, this means that the CAS score plays a major role in the prognostic assessment of ESCC patients than other inflammation-related indicators. Thus, the CAS score composite can be applied to predict the high-recurrence risk of esophageal cancer patients as well as to establish a mechanism for more effective treatment strategies to improve postoperative survival and quality of life of patients.

## Conclusion

The prediction model including the CAS has excellent accuracy, a high net revenue, and favorable prediction function.

### Limitation

There are as yet many drawbacks in this study, such as follows: the sample volume of this report is modest, and the population is homogeneous and non-extensive. Secondly, due to the retrospective character of this investigation and the difficulty of data gathering up to 5 years ago, the comparison of important indicators such as C-reactive protein is lacking in the study, and the content is incomplete. Third, the updated laboratory tests prior to surgery were used to calculate AGS as well as SMI, and they may be influenced by several reasons including preoperative preexisting albumin infusion or blood transfusion and subjective calculation of area error.

## Supplementary Information

**Additional file 1.** Variable model evaluation.

## Data Availability

The datasets used and/or analyzed during the current study are available from the corresponding author on reasonable request.
