# Supplementary material for: A possible combined appraisal pattern: predicting the prognosis of patients after esophagectomy
Source: World J Surg Oncol. 2023 May 22;21:155. doi: 10.1186/s12957-023-03020-x (PMC10201727; doi:10.1186/s12957-023-03020-x)
Supplement: Supplementary file 1 — Additional file 1. Variable model evaluation. [file 12957_2023_3020_MOESM1_ESM.doc]

Additional file 1 Variable model evaluation

| Variable | AUC | Cut-off | Specificity | Accuracy |
| --- | --- | --- | --- | --- |
| Operation duration | 0.52 | 245.0 | 0.51 | 0.52 |
| Bleeding volume | 0.58 | 130.0 | 0.70 | 0.59 |
| Lymph dissection | 0.48 | 13.0 | 0.46 | 0.50 |
| Drainage | 0.54 | 1475.0 | 0.48 | 0.52 |
| PLR | 0.56 | 154.9 | 0.58 | 0.56 |
| NLR | 0.54 | 2.38 | 0.53 | 0.54 |
| SMI |  |  |  |  |
| Female | 0.73 | 32.9 | 0.45 | 0.44 |
| Male | 0.70 | 42.3 | 0.22 | 0.25 |
| CIN | 0.61 | 51.6 | 0.38 | 0.40 |
